# Emerging trends in research and development on earth abundant materials for ammonia degradation coupled with H_2_ generation

**DOI:** 10.1080/14686996.2023.2301423

**Published:** 2024-01-09

**Authors:** Zakiullah Zaidi, Kamlesh, Yesleen Gupta, Sandeep Singhai, Manish Mudgal, Archana Singh

**Affiliations:** a CARS and GM, CSIR-Advanced Materials Process Research Institute (AMPRI), Bhopal, India; b Academy of Scientific & Innovative Research (AcSIR), Ghaziabad, India

**Keywords:** Nitrogen reduction reaction (NRR), ammonia decomposition reaction (ADR), green fuel, catalytic oxidation, non-noble metal

## Abstract

Ammonia, as an essential and economical fuel, is a key intermediate for the production of innumerable nitrogen-based compounds. Such compounds have found vast applications in the agricultural world, biological world (amino acids, proteins, and DNA), and various other chemical transformations. However, unlike other compounds, the decomposition of ammonia is widely recognized as an important step towards a safe and sustainable environment. Ammonia has been popularly recommended as a viable candidate for chemical storage because of its high hydrogen content. Although ruthenium (Ru) is considered an excellent catalyst for ammonia oxidation; however, its high cost and low abundance demand the utilization of cheaper, robust, and earth abundant catalyst. The present review article underlines the various ammonia decomposition methods with emphasis on the use of non-noble metals, such as iron, nickel, cobalt, molybdenum, and several other carbides as well as nitride species. In this review, we have highlighted various advances in ammonia decomposition catalysts. The major challenges that persist in designing such catalysts and the future developments in the production of efficient materials for ammonia decomposition are also discussed.

## IMPACT STATEMENT

In this dynamic area, ammonia degradation to hydrogen fuel provides a valuable contribution in the carbon neutral economy. Ammonia has been used extensively in several industries and is considered an ideal candidate for hydrogen generation and storage due to its high hydrogen content. Consequently, the ammonia decomposition to yield green hydrogen has become a hot topic in research. Although numerous studies on ammonia decomposition have been conducted over the last few decades, still very few review articles on the most recent advances in this field of catalysis have been published. Through this review, systematic information on the types of decomposition catalysts including both noble (Ru) and non-noble earth abundant metals such as iron, nickel, cobalt, molybdenum, their carbides and nitrides, catalytic routes, as well as the reactivity and mechanism can be comprehended. The literature on newly discovered catalysts, specifically from the last five years, is well documented and explained in this review article. Furthermore, the effect of catalyst supports, their reaction kinetics and mechanistic insights have also been discussed. The challenges and opportunities associated with the decomposition catalysts are comprehensively explicated in the end.

## HIGHLIGHTS

1. Ammonia decomposition reaction (ADR) is a viable method for hydrogen storage in the form of chemical bonds.
2. Catalysts composed of noble, non-noble metals, amides, imides, carbides, nitrides, and their combinations have been widely explored towards the ADR.
3. Challenges and opportunities in the ammonia oxidation are pointed out.

## GRAPHICAL ABSTRACT

**Figure:**
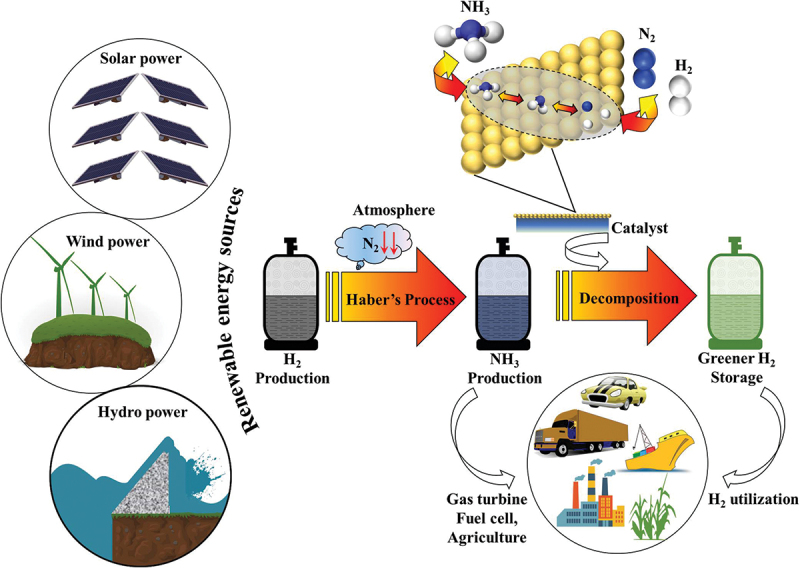

## 1. Introduction

**Figure 1.**
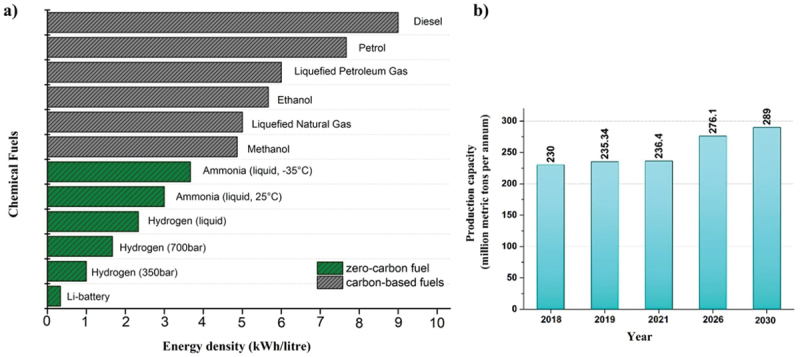
(a) Energy density of a range of chemical fuels. (b) Estimated rise in annual ammonia production.

Owing to the rising demand for energy and depleting fuel reserves, the consumption of valuable fossil feedstocks has become inevitable. Fossil fuels form an important source of a variety of hydrocarbons and energy carrier in the world [1,2]. However, the widely increasing population and excess industrialization have made them a huge polluter [3]. Despite the known damaging effects of fossil fuels, their excellent applications are undeniable. Fossil fuels such as coal, petroleum, and natural gas meet around 90% of the world’s total energy demand [4]. However, their utilization involves the burning process which liberates a significant amount of carbon dioxide to the environment [5]. The achievement of carbon neutrality, in which CO_2_ released into the atmosphere is balanced by an equivalent amount removed, is far too important for a sustainable environment [6,7]. Therefore, there is a serious need to replace fossil fuels with an alternate fuel that must be convenient and as effective. Ammonia is one of the key components released by the combustion of fossil fuels. Globally, more than 70% of ammonia is generated by the burning of fossil fuels [8]. However, it emerges as a key solution towards the clean energy requirement of the world. Ammonia is involved as an important building block for the manufacture of a range of chemicals such as from fertilizers to fuels [9]. According to the reports published by the Royal Society in 2020, ammonia has been regarded as a zero-carbon fertilizer, fuel, and energy store. The average energy density of ammonia is about 3 kWh/litre, which is less than fossil fuels but still comparable in terms of its energy storage properties (Figure 1(a)) [10]. Moreover, the power conservation capacity of ammonia (20.1 MJ/kg) is the same as that of methane (18.6 MJ/kg). The sole difference lies in the oxidation process, while latter releases CO_2_, the former releases N_2_ gas into the atmosphere. The global data estimated that the rise in annual ammonia production from 235 million meter in 2019 up to 290 million metric tons by the end of the year 2030 (Figure 1(b)). This significant rise is due to the enormous population growth and vast growth in the industrialization. Ammonia is considered as a zero-carbon energy vector for the future generation. However, it is not economically viable to store and transport hydrogen on a massive scale in its pure form. In contrast, the use of ammonia as a hydrogen-based synthetic fuel or as a hydrogen template appears to be extremely favourable.

Over the past few decades, ammonia is the world’s second most produced chemical after sulfuric acid (globally over 200 million tons per annum). With expanding potential applications like its utilization in the feed stock and agriculture sector, its world production could rise by several orders of the magnitude. The primary benefit of ammonia is that it contains 40% more hydrogen than methanol and can be produced from renewable hydrogen and nitrogen without the utilization of any carbon-based species [11]. However, the utilization of ammonia for their application in various power technologies offers certain challenges and requirements as provided below (Table 1) [12].

**Table 1.** Fuel-associated ammonia technologies.

S. No.	Efficiency	Required pre-treatment	Per capita cost* (in $/kW)
1.	Proton exchange membrane fuel cell	i) Decomposition of ammonia	100 (mobile)
		ii) Trace content of ammonia removal	1300 (static)
2.	Alkaline fuel cell	None	1300 (static)
3.	Solid oxide fuel cell	None	760 (static)
4.	Internal combustion engine	Partial decomposition of ammonia	30–45 (mobile)
			1000 (static)
5.	Boilers and furnaces	None	150–350 (static)
6.	Combined cycle gas turbines	Partial decomposition of ammonia	750 (static)

*Estimated cost for both mobile and static applications are based on recently developed technologies.

**Figure 2.**
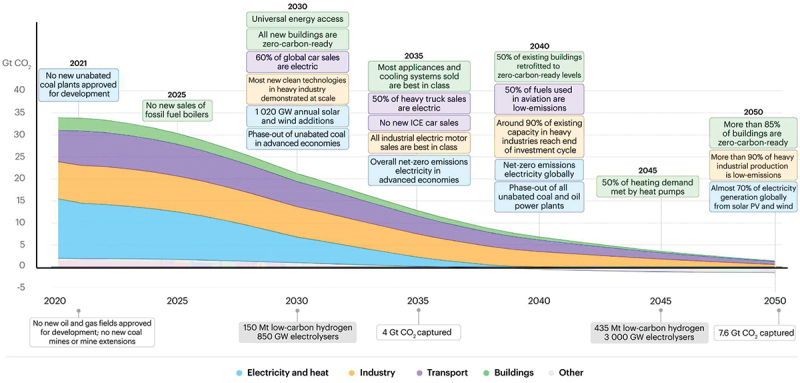
Estimated net zero carbon energy goals from 2020 till the end of the year 2050 (citation: 2020, International Energy Agency (IEA)).

Ammonia is regarded as the most significant option in terms of developing green energy production techniques and simultaneously satisfying the need for sustainable production of chemical fuels or building block chemicals. Moreover, ammonia decomposition reaction (ADR) is regarded as the most environmentally benign, energy efficient, and economically viable step in achieving sustainable future goals. Although ecological awareness has led to the usage of solar energy, geo thermal energy, and hydro energy as a replacement of fossil fuels, their restricted operating conditions limit their practices and hence their execution at individual level will still take time. Considering all these points, the reliable answer is hydrogen which again is directly linked with the production as well as degradation of ammonia gas. As per existing electrolysers technique, the production of 1 kg of H_2_ from fuel combustion causes an amount of 8.8 kg of CO_2_ emission [13]. In contrast to this, the only by-product ammonia degradation generated in addition to H_2_ would be nitrogen gas (N_2_). As a result, no additional handling of greenhouse emissions (GHE) is required [14]. The International Energy Agency (IEA) has proposed a roadmap guide for net zero energy goals by the year 2050 (Figure 2). That means that there would be a huge decline in the use of fossil fuels and the energy sectors would be based on the supply of renewable sources only [15].

The concept of using ammonia as a source of hydrogen is not recent, and the process of cracking or more profoundly called as ammonia decomposition to produce forming gas (H_2_ + N_2_) has long been employed on a vast scale in industries [16]. Breaking of nitrogen-hydrogen bonds in ammonia (NH_3_) results in a net energy gain that affects in the production of nitrogen and hydrogen together with oxygen [17]. Significantly, this indicates that green hydrogen could be produced sustainably using only selective catalyst, assuming sustainable energy is employed to power the operation. For a wide range of applications, hydrogen and nitrogen have the potential to be cost-effective net-zero energy sources and carriers. To more precisely assess this potential, development, demonstration, and deployment are essential.

In this perspective review article, the clean ammonia as a prospective to achieve zero-carbon solution is explored. Furthermore, different types of catalysts which show promising results to achieve hydrogen production are discussed. Considering the fact that a review which covers all types of ammonia degradation catalysts is yet to be reported, the present review article discusses all classes of effective catalysts with their activities and properties.

## 2. Ammonia as carrier for sustainable hydrogen production

**Figure 3.**
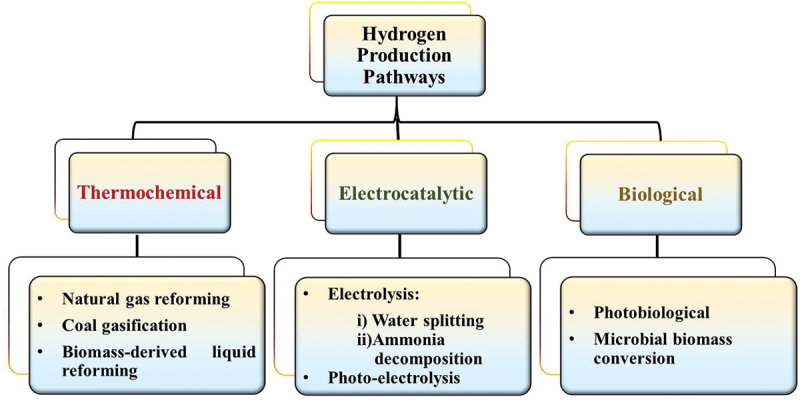
Various hydrogen production pathways.

Hydrogen, as an energy carrier, is becoming increasingly important in achieving a transition to a low carbon of hard-to-abate sectors. Industries, including refining, fertilizers, and steel industries, emit large amount of carbon dioxide and, therefore, carbon-free hydrogen will be essential to allowing deep emissions reductions. Hydrogen is one of the major options being studied with the potential to be the preferred alternative for many applications. Hydrogen can be produced using a number of methods. Different organic as well as inorganic materials such as fossil fuels, biomass, or water release hydrogen gas upon treatment [18]. Some microbial and bacterial species even produce hydrogen through different biochemical ways (Figure 3).

Natural gas reforming, or more commonly known as steam reforming or hydrocarbon reforming process, is one of the most widely used processes by industries on a larger scale for H_2_ production. Here, high-temperature steam, 700–1000°C, is passed through methane gas to produce syngas (CO + H_2_), further through water gas shift reaction, carbon monoxide is converted to carbon dioxide with simultaneous production of large amount of heat [19]. Apart from this, coal gasification and hydrocarbon pyrolysis are other ways of producing hydrogen through fossil fuel combustion [20,21]. An overview of various hydrogen production methods using non-renewable energy sources is provided in Table 2.

**Table 2.** Summary of the basic conditions for H_2_ production through non-renewable sources.

Process	Raw material/ feed stock	Chemical-equation followed	Temperature required (ºC)	By-product	Nature
Steam reforming	Lighter hydro-carbons (LPG or coal)	$CH_{4}+H_{2}O\to CO_{2}\;+H_{2}$	800–1000	CO, CO_2_	Endothermic
Partial methane oxidation	Methane, Heavy fuel oil	$CH_{4}+1/2O_{2}\to \mathrm{CO}\;+2H_{2}$	>1000	CO, CO_2_	Exothermic
Coal gasification	Coal	$C\left(\mathrm{coal}\right)+O_{2}+H_{2}O\to CO_{2}\;+H_{2}$	700–1800	CO, CO_2_	Exothermic
Pyrolysis	Hydrocarbons	$C_{X}H_{Y}\to \mathrm{xC}+\frac{1}{2}yH_{2}$ $C_{X}H_{Y}+\left(2x-\frac{y}{2}\right)H_{2}\to \mathrm{xC}H_{4}$ $CH_{4}\to C+2H_{2}$	500–800	C (solid), CH_4_	Endothermic
Biomass derived liquid reforming	Methanol, Ethanol, Sugars, Acetic acid	$C_{2}H_{5}\mathrm{OH}+H_{2}O\to CO_{2}\;+H_{2}$ $CH_{3}\mathrm{OH}+H_{2}O\to CO_{2}\;+H_{2}$	>800	CO, CO_2_	Endothermic

LPG: Liquefied Petroleum Gas.

However, the other way is to produce hydrogen through the energy provided by renewable sources. In this category, water electrolysis as well as ammonia decomposition are widely recognized processes [22,23]. Low-carbon-footprint hydrogen can minimize CO_2_ emissions and thus limit the global temperature to 2°C. Different technologies based on electro-, photo-, and bio-oxidation for hydrogen production have been provided in Table 3.

**Table 3.** Different hydrogen production technologies from a renewable source of water.

Technology	Source	Temperature required (ºC)	By-product
Electrolysis	Electricity	40–900	O_2_
Photo electrolysis	Light/solar energy	>1200	O_2_
Thermolysis	Heat	>2500	O_2_
Bio-photolysis	Microbes	Ambient conditions	O_2_

Probably, the water splitting from ammonia is considered the most promising way to obtain H_2_ with simultaneous oxygen production. In an overall reaction, two reactions take place in each electrode, the anode and the cathode to convert electrical energy into chemical energy that is released in the form of hydrogen and oxygen as a by-product, Equations (1)–(3).

(1) $$\text{Atanode},H_{2}O\to 1/2O_{2}+2H^{+}+2e^{-}$$

(2) $$\mathrm{Atcathode},\;2H^{+}+2e^{-}\to H_{2}$$

(3) $$\mathrm{Overallreaction},\;H_{2}O\to H_{2}+1/2O_{2}$$

Similarly, solar water splitting, or photolytic, processes the breakdown of water into hydrogen and oxygen using light energy [24]. While still in varied early stages of development, these procedures have the potential to produce sustainable hydrogen in the future with minimal impact on the environment. However, the effectiveness of production is heavily dependent on the geographic setting, i.e. periods of maximum prolonged wind and/or high solar irradiation, and as a result, the produced H_2_ must be stored or carried over extremely long distances to the consumers. Moreover, the storage and transportation of hydrogen faces a number of inherent challenges [25]. H_2_ is frequently liquefied at very low temperatures and under high pressure. Due to the lower density of H_2_ and the relatively huge weight of the carrying vessel, H_2_ still has a low energy density, even at high pressure [26]. Additionally, compression requires 10–13% of the hydrogen’s net energy, making it an energy-intensive process. Hydrogen may be carried as a liquid by cooling it to below 20 K [27]; however, the energy required to liquefy reduces the net energy content (consume approximately by 40%). Additionally, the high combustible range of H_2_ (5–75%) demands extra care while transporting, storage, and use [28]. Hence, considering all such limitations, ADR represents an extremely interesting and promising way for H_2_ production since only N_2_ is generated as a by-product and it can be easily liquified at ambient pressure (8–10 bar). ADR process operates endothermically at slightly high temperature and low pressure, according to Equation (4) [29].

(4) $$2NH_{3}\;\to N_{2}\;+3H_{2}\;\;\Delta H=+45.90\;\mathrm{kJ}/\mathrm{mol}$$

Compared with the water splitting, ammonia decomposition requires substantially less energy to take place since it may spontaneously combine with oxygen to form N_2_O and produce a lot of heat, which powers water splitting [12]. Simultaneous N_2_O breakdown happens when exposed to light. As a result, ammonia may be able to generate hydrogen by utilizing oxygen to promote the breakdown of water.

A catalytic pathway influences ammonia degradation via endothermic reactions and the reverse process of ammonia synthesis. Furthermore, this reaction mechanism entails the entire dehydrogenation process in a stepwise manner, with hydrogen and nitrogen desorption occurring at the end of the reaction. Considering the choice of catalyst which significantly promotes the modification of the strong basic components induces the potential active species and accelerates the nitrogen desorption step via the electron transfer mechanism. In this paper, we reviewed a number of research trends that were carried out by utilising various classes of proficient catalysts for highly efficient ammonia decomposition.

## 3. Ammonia decomposition catalysts

**Figure 4.**
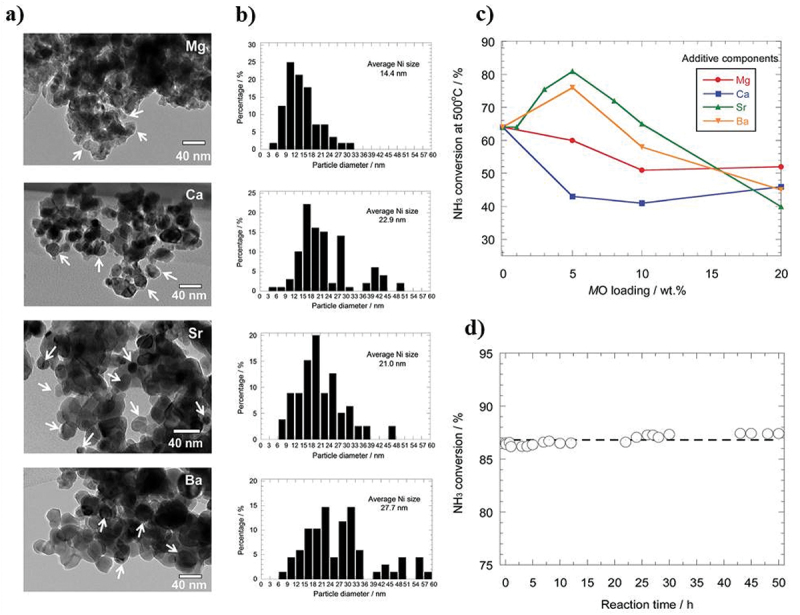
Catalytic activity for ammonia decomposition over alkaline earth metals modified Ni/Y_2_O_3_; (a) TEM images, (b) particle size distribution, (c) NH_3_ conversion percentage, and (d) stability performance of SrO-Ni/Y_2_O_3_ (5:40 wt%), (copyright @ 2016 RSC Advances).

In recent years, researchers have used different materials such as noble metal, non-noble metal, single-atom, and defect materials to obtain higher NH_3_ yield rates and better Faradaic efficiency. Since the decomposition of ammonia is the inverse reaction of the Haber-Bosch process for the synthesis of ammonia, initially the same catalysts which are used for the synthesis, i.e. Ru and Fe, were considered for the thermal decomposition of ammonia assuming the principle of micro reversibility in heterogeneous catalysis [30]. Afterward, Cu-based catalysts were studied as well as other metals [31,32], including Ni [33,34], Ir [35,36], Mo [37], Co [14,38,39], Pt [40], Pd [41], Ru [42,43] and Rh [44] and different combinations of metals and non-metals [45]. The effect of alkaline earth metals (Mg, Ca, Sr, and Ba) over Ni/Y_2_O_3_ catalysts for ammonia decomposition has been studied, with remarkable results achieved for Sr and Ba modification and no significant results obtained with Mg and Ca [45]. The catalytic performance was improved upon reaching at 500°C, their physical characterization, percentage conversion, and stability test of prominent catalyst have been shown in Figure 4.

Ruthenium supported on various oxides or structured and unstructured carbon has been reported to have the highest catalytic activity for the breakdown of ammonia among the catalysts investigated in the literature, with deactivation being the main issue. Ruthenium, however, is a rare, expensive noble metal that is rare in nature. This is why inexpensive catalytic compositions with catalytic activity similar to ruthenium’s have been aggressively sought after.

### 3.1. Noble metals as ammonia decomposition catalysts

The resistance of the noble metals to oxidation and corrosion is used to classify them. Although there is no clear definition for them, Ru, Rh, Pd, Ag, Re, Os, Ir, Pt, and Au are frequently taken into consideration [46]. However, ruthenium is a noble metal that has been the focus of most research as a catalyst for ammonia oxidation.

#### 3.1.1. Ruthenium (Ru)

**Figure 5.**
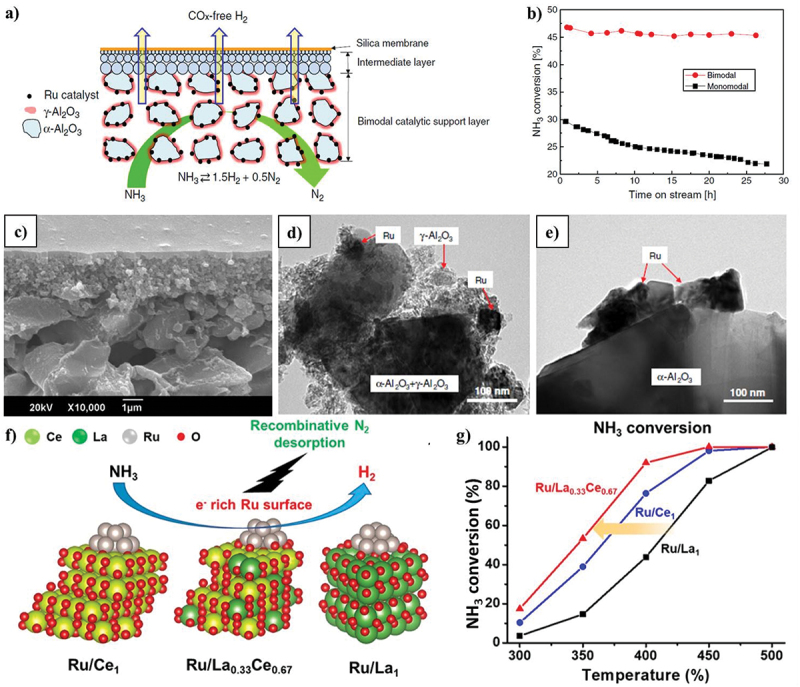
Incorporation of catalytic membrane for ADR process. (a) Schematic catalytic membrane reactor, (b) catalytic activities, (c) TEM image of bimodal, (d) TEM image of monomodal, and e) SEM image of bimodal catalytic membrane (copyright @ 2011 Elsevier). (f) Mechanistic interaction of Ru doping in La_x_Ce_1-x_O_y_ composites and (g) catalytic performance by varying different temperature for NH_3_ conversion (copyright @ 2021 Elsevier).

The catalyst Ru/CNTs (ruthenium supported on carbon nanotubes) promoted with Cs (20 weight percent) was described by Hill and Torrente-Murciano as the one thought to be the most active to date in the decomposition of ammonia [47]. In a subsequent investigation, they prepared the same catalysts with the carbon nanotubes graphitized and with a reduced Cs concentration (4 wt%). Under the same reaction conditions, they obtained a considerable increase in conversion compared to the catalyst with the support without graphitization. It is important to note that nitrogen doping modifies the characteristics of carbon and further boosts the catalyst’s catalytic activity [48]. Comparing Ru supported on ordered mesoporous carbon (OMC) doped with nitrogen to other types of carbon (CNTs, activated carbon, or undoped OMC), the former showed a high catalytic activity [49]. Generally speaking, carbon nanofibers are shown to be the best support for Ru, showing comparable catalytic activity to Ru/CNTs [50]. In this work, the conversion of NH_3_ increases noticeably (from 45% to 95%) at 450°C, when the membrane is used. A schematic bimodal and monomodal catalytic membrane, catalytic activities, and their morphologies have been represented in Figure 5(a–e).

Ru-based catalyst’s simplicity has been shown to be crucial. Bajus et al. investigated to see if Ru/Al_2_O_3_ could be promoted using the same alkali metals that exhibited a promoting effect on Ru supported on carbon [51]. The three metals Li, Na, and Cs are said to be able to help the catalyst, according to their report. The turn-over frequency (TOF) quantifies the activity of the catalytic centre by utilizing the number of molecular reaction or catalytic cycles occurring at the centre per unit time. The TOF for Na and Cs was around double that of the unpromoted catalyst, with Li showing the greatest increase (0.26 vs 0.05 s^−1^ at 350°C). Sayas et al. found that CaO, the most fundamental support of the materials utilised, produced the best catalytic activity when comparing catalysts based on Ru promoted with K supported on different materials (CNTs, MgO, and CaO) [33]. In comparison to two extensively used supports in the literature, carbon nanotubes and SiO_2_, the Ru supported on a layered oxide consisting of Ca and Al (CaAlO_x_) had a higher conversion (78 vs 21 and 62% at 450°C, respectively) [52]. Similar to this, Hayashi et al. compared the outcomes with conventional catalysts of Ru supported on CaO, Al_2_O_3_, and MgO and evaluated electride, [Ca_24_Al_28_O_64_]^4+^(O^2-^)_2_, and mayenite oxide as supports [53]. They also made catalysts for Ru/MgO and Ru/C stimulated by K and Cs, respectively. The catalyst supported on [Ca_24_Al_28_O_64_]^4+^(e^−^)_4_ demonstrated the best conversion at comparatively low temperatures (400°C). When added to an alumina support, barium has also demonstrated its ability to promote catalytic activity. For instance, compared to conventional supports such as MgO, Al_2_O_3_, and CNTs, Ru supported on barium hexaaluminate (BHA) has enhanced catalytic activity that is approximately 4 times higher than MgO and more than double that of carbon nanotubes. Pure ammonia at 450°C was used and reported to have extremely good stability for 60 h [54]. Nagaoka et al. conducted a thorough investigation of the catalytic activities of Ru supported on Pr_6_O_11_. The promoted Pr_6_O_11_ underwent ammonia conversion in the order Cs_2_O > Rb_2_O > K_2_O > Na_2_O, where Cs_2_O is the most fundamental oxide [55]. The ammonia conversion of Ru/Pr_6_O_11_ enhanced by Cs_2_O was extremely high. They found that the non-promoted Ru/Pr_6_O_11_ catalyst has comparable catalytic activity to Ru supported on other oxides, such as Ru/La_2_O_3_. Recently, Thien An Le et al. reported a co-precipitated procedure for synthesising a noble catalyst for the low-temperature decomposition of ammonia by doping Ru in La_x_Ce_1-x_O_y_ (Figure 5(f)) [56]. The results show that the prepared Ru composite demonstrated the best catalytic performance at temperatures below 400°C (Figure 5(g)) by maintaining its stability performance for more than 100 h.

In addition to it, iridium, palladium, platinum, and rhodium are other noble metals that have been examined in the nitrification of ammonia [57–60]. For instance, Maeda et al. demonstrated higher catalytic activity utilising Rh/SiO_2_ boosted with niobium (Nb) compared to a catalyst made of Rh supported on Nb_2_O_5_ [61]. Richardson et al. examined platinum supported on alumina and found limited catalytic activity [62]. Table 4 lists some catalysts based on ruthenium and other noble metals with their catalytic performances towards ammonia decomposition.

**Table 4.** Catalysts based on noble metals utilization to decompose ammonia.

Active phase	Supported on	% NH_3_ conversion	References
Ru	@La_2_O_3_−ZrO_2_	34	[63]
Ru	@La_2_O_3_−ZrO_2_	81	[64]
Ru	@SiO_2_	63	[64]
Ru	@SiO_2_	96	[63]
Ru	[Ca24Al28O64]^4^+(O^2^−)2	73	[65]
Ru	[Ca24Al28O64]^4^+(e−)4	42	[53]
Ru	AC	70	[53]
Ru	AC	25	[66]
Ru	AC	4	[67]
Ru	AC	5	[68]
Ru	Al_2_O_3_	7	[69]
Ru	Al_2_O_3_	13	[53]
Ru	Al_2_O_3_	37	[70]
Ru	Al_2_O_3_	7	[54]
Ru	MgO	39	[71]
Ru	MgO	29	[72]
Ru	MgO	76	[73]
Ru	MgO-Al_2_O_3_	86	[74]
Ru	MgO-MIL-101	45	[75]
Ru	MgO-MIL-101	88	[75]
Ru	Mg_y_AlzO_n_	17	[76]
Ru	MIL-101	42	[75]
Ru	MIL-101	66	[75]
Ru	MWCNT	31	[71]
Ru	Na_2_Ti_3_O_7_	86	[77]
Ru	N-CNTs	48	[78]
Ru	N-CNTs	85	[77]
Ru	N-OMC	69	[78]
Ru	O-CNFs	59	[55]
Ru	OMC	41	[55]
Ru	Pr_6_O_11_	20	[55]
Ru	Pr_6_O_11_	19	[55]
Ru	Pr_6_O_11_	49	[55]
Ru	Pr_6_O_11_	27	[55]
Ir	Al_2_O_3_	86	[79]
Ir	Al_2_O_3_	45	[80]
Ir	SiO_2_	88	[80]
Pd	SiO_2_	17	[81]
Pd	Al_2_O_3_	42	[79]
Pd	SiO_2_	31	[81]
Pt	Al_2_O_3_	86	[79]
Pt	Al_2_O_3_	61	[62]
Pt	MCM- 41	85	[82]
Pt	SiO_2_	69	[82]
Rh	Nb_2_O_5_	20	[61]
Rh	SiO_2_	19	[61]

### 3.2. Non-noble metals as ammonia decomposition catalysts

Nickel is the non-noble metal that has received the most attention among those utilized as catalysts for the breakdown of ammonia because of its extraordinary activity. Once more, the catalytic performance is significantly impacted by the type of support. When comparing the transition metals Ni, Fe, and Co distributed in an alumina matrix, Gu et al. found that the cobalt catalyst had the highest catalytic activity, followed by the Ni catalyst [83], while the Fe/Al_2_O_3_ catalyst had the lowest catalytic activity. After more than 70 h, all three catalysts demonstrated high stability for the degradation reaction. The catalysts supported on MgO modified with La and on CNTs produced the same order of catalytic activity. Similar to this, Yan et al. found that the Co catalyst has a higher activity (85 vs. 30% conversion at 500°C) when comparing the breakdown of ammonia in the presence of Fe and Co catalysts [84]. On the other hand, both catalysts displayed a decline in activity following a second reaction cycle, despite the fact that the addition of lanthanum to the catalyst improved both catalysts’ stability. When comparing the catalytic outcomes of several low-ordered carbon types with and without the addition of Fe or Ca, Xu et al. demonstrated that adding Fe to the catalyst can boost conversion while adding Ca has a negative impact on the reaction.

#### 3.2.1. Monometallic catalysts

There have been numerous monometallic catalysts; however, few of them such as nickel, iron, cobalt, and molybdenum have been widely utilized in their active phase for ammonia decomposition reaction [14].

##### 3.2.1.1. Nickel (Ni)

As was already indicated, the industrial ammonia crackers use commercially available catalysts that are largely made of nickel supported on alumina. According to the research given by Zhang et al., the best TOF with Ni/Al_2_O_3_ catalysts is produced with metallic Ni particles ranging in size from 1.8 to 2.9 nm [85], and doping alumina with lanthanum boosts the catalytic activity. In this study, La could partially reduce the particle size of Ni^0^ and thus influence the kinetic results of Ni/Al_2_O_3_ and Ni/La-Al_2_O_3_ catalysts at 733–813 K. In this regard, Yan et al. developed a porous microsphere catalyst made of Ni, Ni_0.5_Ce_0.5_O_x_, Ni_0.5_Al_0.5_O_x_, or Ni_0.5_Ce_0.1_Al_0.4_O_x_, and concluded that the catalyst made of nickel and cerium performed better than Ni_0.5_Al_0.5_O_x_, while Ni_0.5_Ce_0.1_Al_0.4_O_x_ increased the stability of the catalyst [84]. Rare earth promoters seem to have a good impact on Ni’s catalytic activity. Okura et al. discovered that the promoting effect on the Ni/Al_2_O_3_ catalyst followed the path La > Pr > Nd > Y > Sm > Eu Gd > Ce [86].

Silica has also been studied as a support for nickel catalysts in a variety of forms, including SiO_2_, mesoporous frameworks, and as a natural mineral [87]. Choudhary et al. examined a variety of Ni supported on different silica and zeolite types (HY and H-ZSM-5) as well as a catalyst based on a silica and alumina combination, resulting in the following catalytic activity rankings: Al_2_O_3_-SiO_2_ > SiO_2_~HY > H-ZSM-5. At temperatures below 650°C, it was discovered that the catalyst with the lowest pore diameter (7.7 nm) displayed the maximum activity, however at higher temperatures, the conversion was higher with wider pores as a result of internal mass transfer problems. Zirconium-based supports are another class of substances that have been tested as Ni supports. When ZrO_2_ was evaluated as a dopant for an alumina support, for instance, Henpraserttae et al. found that the catalyst’s ammonia conversion increased by 11% at 500°C compared to the catalyst supported exclusively on alumina. When compared to a catalyst that had the promoter supplied directly to the active Ni phase, the catalyst supported on the oxide mixture demonstrated greater catalytic activity. A larger dispersion of Ni and an increase in basic sites are credited with the Zr-doped sample’s improved activity [88,89].

##### 3.2.1.2. Iron (Fe)

**Figure 6.**
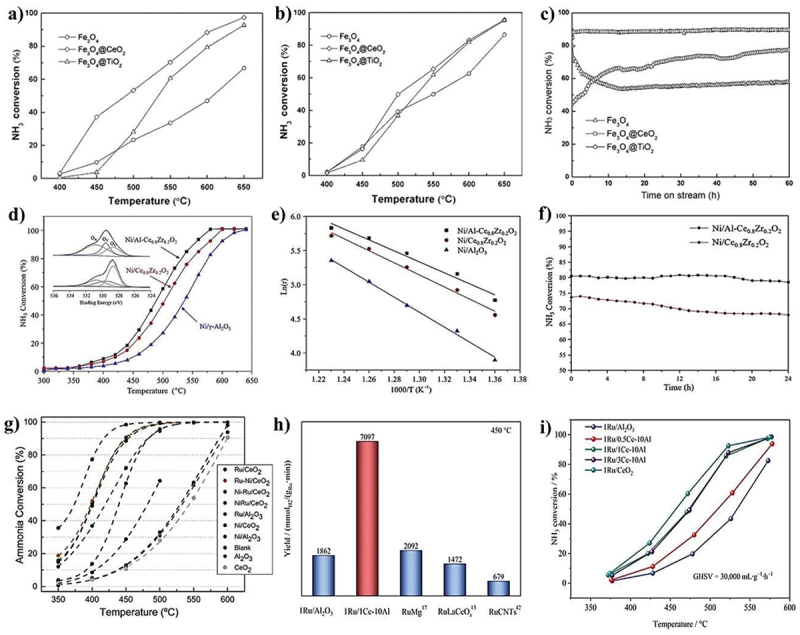
Varying different temperature for NH_3_ conversion over iron-based nanostructured composites (a) 1st catalytic performance, (b) 2nd catalytic performance, and (c) stability performance (copyright @ 2015 Elsevier). Varying different temperature for NH_3_ conversion over ceria-based oxide supported nickel composites (d) catalytic performance, (e) Arrhenius plot, and (f) stability performance (copyright @ 2020 Elsevier). (g) Varying different temperature for NH_3_ conversion over ceria-based oxide supported Ni, Ru, and Ni-Ru composites (copyright @ 2019 Elsevier), varying different temperature for NH_3_ conversion over ceria-based oxide supported Ru/Al_2_O_3_ composites. (h) Comparative study with literature reports and (i) catalytic performance (copyright @ 2023 Elsevier).

A natural iron mineral with impurities of TiO_2_, CaO, Al_2_O_3_, K_2_O, SiO_2_, and Mn made up a catalyst that had strong activity but was unstable over time, with a quick decline in a 3 h test. An exclusively made of Fe_2_O_3_ reference catalyst produced the same behaviour. Tseng et al. asserted through in-situ studies that the active form of catalysts made of Fe is Fe_3_N_x_, while FeN_x_ is generated at high temperatures (>675°C), which has a detrimental effect on the conversion of ammonia [90]. At 500°C, a Fe_2_O_3_ catalyst based on SBA-15 mesoporous silica has higher catalytic activity than iron oxide alone (18 vs. 4%). Fe nanoparticles encased in silica are significantly more active (conversion of 9 vs 27% at 500°C), according to research by Li et al. It has been shown that Fe is substantially more stable for the reaction when it is encapsulated in SiO_2_. Li et al. found that adding Cs to this catalyst increased the ammonia conversion at 450°C by about twofold compared to the catalyst without promoting [91]. Additionally, under the same reaction conditions, the catalytic activity increased when Fe nanoparticles were enclosed in Al_2_O_3_ (9 vs. 4% at 450°C). Cui et al. evaluated the activities of iron oxide alone and iron oxide modified with cerium or titanium oxide, finding that the composite catalysts produced a higher and more stable conversion over time [92]. In this study, iron-based nanostructured composites were utilized for NH_3_ decomposition in a fixed-bed continuous-flow quartz reactor by varying different catalytic temperatures (400–650°C) (Figure 6(a,b)). The composite catalyst has high stability when heated by ramping the temperature to 600°C for 60 h, shown in (Figure 6(c)). Their morphologies of fresh and used Fe_3_O_4_/composites show spherical shapes with average particle sizes of 100 nm. It is important to note that the modification using ceria was the most successful. Compared to nickel, Fe and carbon-based supports work better together. In comparison to Ni/Al_2_O_3_ and Fe supported on a mixture of carbon and SBA-15, supported on ordered mesoporous carbon CMK-5, Fe demonstrated better catalytic activity (74 vs. 32 and 29% at 600°C, respectively). When graphitized carbon (GC) is employed as a support for Fe, great activity and stability can also be attained. Furthermore, when potassium (K) is introduced to Fe catalyst supported on GC, the rate of ammonia decomposition increases [93]. It has been concluded that the study based on ceria, which has rich oxygen vacancies, and its oxides based supported metal composites has a positive impact on the reduction, dispersion, and stabilisation in NH_3_ decomposition, as demonstrated by the various catalytic performance shown in the (Figure 6) [70,94,95].

##### 3.2.1.3. Cobalt (Co)

Despite being less active than iron and nickel, cobalt has little effect on ammonia decomposition. The composite with alumina, on the other hand, increases the catalytic activity [96]. In this work, Co_3_O_4_-Al_2_O_3_ demonstrated excellent catalytic performance towards NH_3_ decomposition, achieving 100% conversion at 600°C and remaining its stability for 72 h. Al, Ca, and K oxides were added as promoters by Czekajlo and Lendzion-Bieluń to boost the conversion [97]. Promoter oxides are thought to stabilise the catalyst’s surface area when they are added. Consequently, by incorporating alumina (10 wt %) into cobalt oxide, the conversion of ammonia was greatly boosted. Gu et al. also achieved a similar outcome using a catalyst of the same composition; the conversion increased by around 20% in comparison to pure cobalt oxide [98].

**Figure 7.**
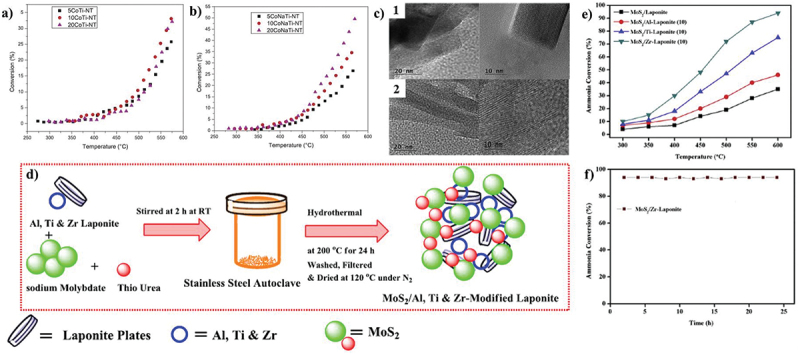
NH_3_ decomposition by (a) ion exchange method, (b) NaOH deposition method, (c) TEM images of **1**. 10CoTi-NT **2**. 10CoNaTi-NT (copyright @ 2019 Elsevier). (d) Synthesis scheme of MoS_2_ catalysts, (e) catalytic activity of MoS_2_ composites, and (f) stability performance (copyright @ 2020 Elsevier).

Co was also investigated and demonstrated good catalytic activity when supported on carbon nanotubes. Multi-walled carbon nanotubes (MWCNTs) outperformed activated carbon (AC), reduced graphene oxide (rGO) and single-walled carbon nanotubes (SWCNTs) in contrast to other carbon supports for Co [99]. Boosting impact of Na on a cobalt catalyst supported on titania nanotubes was demonstrated by Lara-Garcia et al. They found that the catalyst with Co particles of 15 nm had the best catalytic results, which is consistent with literature that claims that Co nanoparticles between 10 and 20 nm in size exhibit the highest activity [100]. In this study, cobalt-supported titanate nanotubes were used as a catalyst for ammonia decomposition followed by different approaches, where the catalytic activity increased as the cobalt particle size decreased (Figure 7(a–c)). Similarly, the boosting impact of La on the activity of Co supported on MgO was also confirmed by Hu et al. At 400°C, the conversion of pure ammonia increases by 12% compared to the catalyst. Hu et al. also evaluated a Co catalyst supported on SiO_2_ [101]. When silicates are utilised as a support, Co exhibits good activity. In this regard, the ammonia conversion was significantly boosted by the incorporation of cobalt into a structured mesoporous silicate. Cobalt included in a sodium silicate structure also exhibited strong activity [39].

##### 3.2.1.4. Molybdenum (Mo)

Mo is another non-noble metal that has been investigated for its ability for various environmental remediation activities such as sulfur removal, hydrogen production, and ammonia degradation [102–105]. Testing catalysts with MoS_2_ as the active phase supported on laponite and laponite modified with Al, Ti, and Zr, Krishnan et al. found that the support changed with Zr produced the best results, achieving a conversion of 94% at 600°C (Figure 7(d–f)). They arrived at the conclusion that adding heteroatoms to the support allows for an improvement in the catalyst’s basicity and MoS_2_ dispersion [106]. Mo_2_C was optimized and catalytically examined by Zheng et al. When using pure ammonia at a high temperature (600°C), the catalyst tended to produce MoN, which resulted in a considerable decrease in specific surface area [107]. Nevertheless, the catalyst had lower activity than Ru supported on graphite. Similar to this, Li et al. evaluated catalysts made of MoO_2_ and Mo_2_C nanoparticles dispersed in a porous carbon matrix, noting that they quickly produced MoN during reaction, which ended up being the active phase [108]. A list of non-noble ammonia-decomposition catalysts as the active phase along with their catalytic performances is provided in Table 5.

**Table 5.** Efficiency of reported non-noble metal catalysts for ammonia-decomposition.

Active phase	Supported on	% NH_3_ flow	% NH_3_ conversion	References
Ni	@SiO_2_	100	36	[63]
Ni	@SiO_2_	100	40	[63]
Ni	@SiO_2_	100	87	[109]
Ni	@SiO_2_	100	47	[63]
Ni	AC	0.2	2	[110]
Ni	AC	15	75	[111]
Ni	Al_2_O_3_	100	15	[85]
Ni	Al_2_O_3_	100	15	[112]
Ni	Al_2_O_3_	100	17	[113]
Ni	Al_2_O_3_	100	97	[114]
Ni	Al_2_O_3_	100	33	[115]
Ni	Al_2_O_3_	100	27	[89]
Ni	Al-Ce_0.8_Zr_0.2_O_2_	100	58	[94]
Ni	attapulgite	100	90	[116]
Fe	@ Al_2_O_3_	100	9	[117]
Fe	@CeO_2_	100	70	[92]
Fe	@SiO_2_	100	8	[91]
Fe	@SiO_2_	100	17	[91]
Fe	@SiO_2_-Cs	100	16	[91]
Fe	@TiO_2_	100	60	[92]
Fe	AC	0.2	90	[111]
Fe	Al_2_O_3_	100	–	[79]
Fe	Al_2_O_3_	50	25	[118]
Fe	Al_2_O_3_	100	86	[83]
Fe	C/SBA-15	100	32	[113]
Fe	Carbon	0.2	96	[119]
Fe	CMK-5	100	74	[113]
Fe	CNFs	100	51	[120]
Co	AC	100	34	[121]
Co	AC	100	2	[122]
Co	Al_2_O_3_	100	–	[79]
Co	Al_2_O_3_	100	21	[123]
Co	Al_2_O_3_	100	100	[83]
Co	Al_2_O_3_	100	44	[124]
Co	Al_2_O_3_	100	57	[98]
Co	AX-21	100	25	[125]
Co	AX-21	100	3	[125]
Co	Ce_0.6_Zr_0.3_Y_0.1_O_2_	100	7	[126]
Co	CeO_2_	100	30	[126]
Co	CNTs	100	100	[127]
Co	CNTs	100	61	[128]
Co	CNTs	100	–	[129]
Co	CNTs	100	–	[130]
Co	CNTs	100	8	[131]
Co	CNTs	100	9	[122]
Mo	Al_2_O_3_	100	22	[123]
Mo	C	100	66	[108]
Mo	MCM-41	100	28	[132]
Mo	Y_2_O_3_-ZrO_2_	100	10	[133]
MoN	C	100	82	[108]
MoN	SBA-15	100	69	[134]
MoN	SiO_2_	100	50	[134]
MoN	Al_2_O_3_	100	99	[135]
MoS_2_	Al-laponite	10	46	[106]
MoS_2_	Laponite	10	35	[106]
MoS_2_	Ti-laponite	10	74	[106]
MoS_2_	Zr-laponite	10	94	[106]
Mo_2_C	–	100	–	[136]
Mo_2_C	–	100	77	[107]
Mo_2_N	–	100	94	[137]
Mo_2_N	–	100	10	[138]
Mo_2_N	–	100	100	[139]

##### 3.2.1.5. Other metal amides, imides, carbides, and nitrides

Choi et al. investigated different vanadium carbide forms and compared their activity to that of Mo_2_C and found that while it was consistently lower than that of the molybdenum catalyst, it was significantly higher than a catalyst made of platinum supported on carbon. In a different study, Choi et al. contrasted the vanadium and molybdenum carbides and their equivalent nitrides, VN and MoN, activities [136]. The outcomes demonstrated that the two element carbides are more effective at causing ammonia to break down.

Alkali metal amides, such as LiNH_2_, KNH_2_, and NaNH_2_, have emerged as promising compounds for the decomposition of ammonia, providing conversion rates that are on par with or better than those of ruthenium-based catalysts [140]. For instance, compared to the Ru/Al_2_O_3_ and Ni/SiO_2_Al_2_O_3_ catalysts under the same catalytic conditions, lithium amide had a significantly higher activity [141]. It was discovered that the active phase of amide catalysts varies depending on the reaction circumstances and the alkali metal of choice. The imide form, NH, of Li amides is the active phase in this scenario. Lithium imide was synthesised by Makepeace et al., adjusted with Ca and Mg, and the outcomes were compared to a lithium amide-imide mixture [142,143]. In comparison to the unmodified catalyst, they noticed that the changed forms exhibit a higher conversion at low temperatures. Additionally, Wood and Makepeace examined the compatibility of the lithium amide-imide catalyst with a number of support materials, including silicon dioxide (SiO_2_), aluminium oxide (Al_2_O_3_), magnesium oxide (MgO), and activated carbon [143]. In contrast to the unsupported lithium amide-imide, there was no improvement in the catalytic performance for any of the configurations [144]. The catalytic activity of LiNH_2_ alone versus supported by carbon was studied by Bramwell et al. Due to the production of Li_2_NCN, no activity was seen with the assisted catalyst. However, the reaction can be delayed until 450°C by adding Ni to the supported catalyst [145]. As a result, compared to LiNH_2_, the catalytic activity of the LiNH_2_/Ni/C catalyst was greater. These results show a 26% greater conversion to LiNH_2_/Ni/C at 400°C under the reaction conditions examined as compared to those achieved using a Ru catalyst supported on alumina. Guo et al. investigated the catalytic activity of MnN mixed with the imide Li_2_NH in this context and contrasted the results with a Ru/CNTs catalyst, getting a greater activity with the MnN-Li_2_NH catalyst under the identical reaction conditions [130]. When combined with the imide CaNH, the nitride Mn_6_N_5_, which is nearly inactive in the degradation of ammonia, demonstrated rather strong catalytic activity. By combining amides with ruthenium, several kinds of catalysts have been synthesized, often utilising mechanochemical processes. Examples include the amides of the alkaline earth metals Ba, Ca, and Mg. A thorough investigation into the catalytic activity of catalysts made of lithium imides and transition metal nitrides (TMN) was conducted by creating a number of catalysts from the corresponding transition metal chlorides and LiNH_2_, then nitriding them one after the other using NH_3_. In comparison to Fe_2_N, Li_2_NH, and Fe supported on CNTs, the Li_2_NH-Fe_2_N catalyst showed a greater ammonia decomposition rate [146]. A list of different metal-based amides, imides, carbides, and nitrides is provided in Table 6 for their catalytic activity in the degradation of ammonia.

**Table 6.** Catalytic activity of metallic amides, imides, carbides, and nitrides.

Active phase	% NH_3_ inlet flow	% NH_3_ conversion	References
CrN	100	0	[130]
Fe_2_N	100	4	[147]
Fe_2_N	100	–	[130]
Fe_3_C	100	23	[148]
MnN	5	15	[149]
WC	100	22	[137]
V_8_C_7_	100	–	[136]
K_2_[Mn(NH_2_)_4_]	5	48	[149]
Li_2_Ca(NH)_2_	100	48	[142]
Li_2_Mg(NH)_2_	100	40	[142]
Li_2_NH	5	0	[73]
Li_2_NH	100	26	[130]
Li_2_NH-Co	–	–	[130]
Ru-KNH_2_	10	96	[140]
Ru-LiNH_2_	5	100	[73]
Ru-Mg(NH_2_)_2_	100	3	[150]
Ru-NaNH_2_	10	97	[140]

#### 3.2.2. Bimetallic and multimetallic systems

Permutations of the metals Co, Mo, Ni, Fe, Pt, Cu, Ir, Cr, Mn, Mg, Cu, Sn, Zn, Li, and Pd have been examined as bimetallic and multimetallic catalysts for the ammonia decomposition reaction [151,152]. Additionally, studies of combinations of Ru with Fe, Sr, Bi, Pb, Sn, In, Cd, Zn, Au, Ag, Cu, Pt, Pd, Ni, Ir, and Rh have been established [153]. However, because of its outstanding catalytic activity, the cobalt and molybdenum combination is one of the most explored bimetallic catalysts. Duan et al. confirmed that Co_3_Mo_3_N was the active phase of the bimetallic catalyst by evaluating an unsupported Co-Mo catalyst [154]. Compared to the monometallic form, Mo_2_N, the bimetallic Co_3_Mo_3_N catalyst, demonstrated greater conversion. Additionally, the monometallic nitride, Mo_2_N [155,156], was outperformed by a catalyst made of Fe_3_Mo_3_N, which was synthesized by nitriding FeMoO_4_. Following up on the earlier findings, Srifa et al. [157] evaluated bimetallic catalysts made by nitriding the appropriate oxides with NH_3_ at 350°C utilising Mo coupled with Co, Ni, and Fe. They listed the catalyst activity in the following order: Co_3_Mo_3_N, Ni_3_Mo_3_N, Fe_3_Mo_3_N, and Mo_2_N.

Bimetallic catalysts have been tested to boost their catalytic activity and to be able to minimise their presence in the catalyst in addition to replacing ruthenium or other noble metals. In this regard, McCullough et al. used a high-throughput technique to test more than 100 bimetallic catalysts based on Ru metal K supported on Al_2_O_3_ for the low-temperature decomposition of ammonia [153]. Similar to this, Dasireddy and Likozar created a bimetallic catalyst made of Cu and Zn supported on alumina and showed that it had better catalytic activity than the corresponding monometallic catalysts [158].

**Figure 8.**
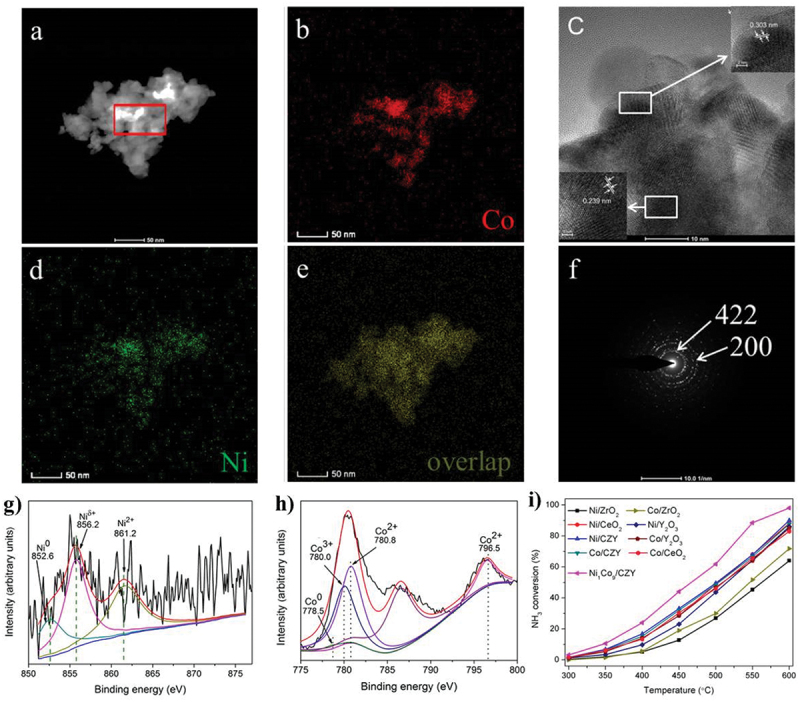
Ammonia decomposition over bimetallic Ni- and Co-based catalyst (copyright @ 2019 Elsevier).

Xie et al. tested the usage of carbon nanofibers (CNFs) as supports. The catalytic activity of the nanofiber in combination with a Co-Mo-Fe-Ni-Cu alloy was compared to that of Ru and a Co-Mo bimetallic catalyst utilizing the same support [46]. By adjusting the proportions of Co and Mo in the composition, they were able to achieve a TOF that was 20 times higher than that obtained with Ru and an even greater increase in TOF when compared to the traditional Co-Mo catalyst. In a 50 h test at 500°C, this multimetallic formulation demonstrated good stability. The catalytic activity of many bimetallic formulations has also been tested using various ceramic supports. For instance, on various oxide substrates, Huang et al. compared the catalytic activity of bimetallic catalysts Ce_0.6_Zr_0.3_Y_0.1_O_2_ (CZY) solid solutions supported with Ni and Co [126]. The NiCo_9_/CZY morphologies, corresponding elemental mapping, and SAED pattern have been shown in Figure 8(a–f), whereas the XPS spectra of the reduced Ni_1_Co_9_/CZY shown in Figure 8(g,h). The physical characterization reveals that the lattice spacing of 0.303 nm corresponds to the (422) planes of Co_3_O_4_. In this study, a steady-state reaction was carried out using Ni- and Co-based catalysts, with Ni/CeO_2_, Ni/Y_2_O_3_, Co/CeO_2_, Co/Y_2_O_3_, Ni/CZY, and Co/CZY showing similar catalytic activity while Ni/ZrO_2_ and Co/ZrO_2_ show lower activity than others, as shown in Figure 8(i). The supports catalytic activity was obtained in the series Ce_0.6_Zr_0.3_Y_0.1_O_2_ > CeO_2_ > Y_2_O_3_> ZrO_2_.

**Figure 9.**
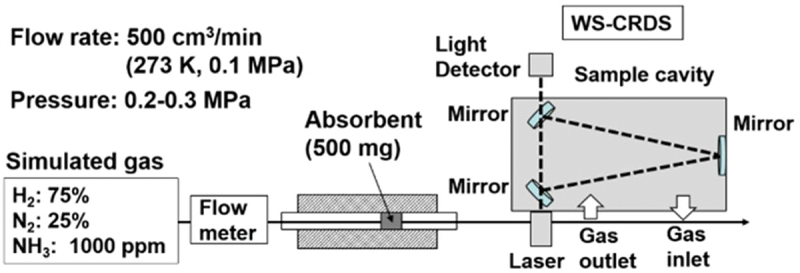
Schematic illustration of PEM fuel cell for H_2_ production from ammonia (copyright @ 2018 Elsevier).

There is currently widespread commercial use of ammonia decomposition, but storing the by-products still poses significant difficulties for the development of a sustainable hydrogen economy. Due to several drawbacks that have hampered the development of hydrogen storage, researchers believe that much more work is required to reach a satisfactory level for commercialization. Although several technologies have been investigated for hydrogen storage, fuel cell transformation is an alternative to utilising hydrogen that directly produces electric energy by combining the electrochemical reaction of hydrogen and oxygen. Since the catalytic route to completely decompose ammonia into hydrogen is quite limited, Miyaoka et al. reports on the reduction of ammonia concentration during decomposition using a polymer electrolyte membrane (polymer electrolyte membrane) fuel cell equipped with Li-exchange X-type zeolite (Li-X) as adsorbent [159]. In this study, the ammonia decomposition reaction was carried out at 773–823 K and 0.1 MPa had to be reduced to less than 0.1 ppm on specially designed apparatus (PICCARO G2103) outfitted with an agate mortar, as shown in (Figure 9).

## 4. Conclusion and future outlook

Ammonia is utilised for a variety of industrial purposes, including plastics, explosives, and synthetic fibres, accounting for around 70% of its use in fertilisers. Ammonia as a fuel offers promise in the context of sustainable energy transitions, however this application is still in its infancy. The focus of this current emphasis is primarily on ammonia’s current use in agriculture and industry. While improvements have been made to existing technologies and new alternatives to fossil fuels have been added over time, a fundamental shift in the energy system, the so-called ‘energy shift’ has yet not occurred. The steep drop in the price of energy storage technologies, as well as solar and wind technologies, is a major driving force behind the planned fundamental change in the energy transition toward a new clean and efficient system. Building on existing initiatives, international collaboration, including on infrastructure, can assist improve the results provided here. Numerous investigations have concentrated on identifying the decomposition reaction’s limiting step utilising various types of catalysts. Although at first it was suggested that the limiting step was the desorption of nitrogen from the catalyst’s surface and that, consequently, the binding energy with nitrogen could determine the activity of the catalyst, it was later discovered that the limiting step was different for different types of catalysts. The reaction is significantly influenced by the active phase, the support, and the promoters. According to conventional wisdom, a catalyst support for NH_3_ decomposition should have a high basicity, high conductivity, low concentration of electron-withdrawing groups, high thermal stability, and high surface area. Numerous studies have specifically linked basicity to catalyst activity, and this relationship has been noted for a variety of active phases including Ru, Ni, Fe, Co, and Mo. Research should be combined with international collaboration to spur further development. For an accurate design of catalytic devices as well as for a better understanding of the reaction process, kinetic studies, and reactor modelling are helpful and essential methods.
